# Characterization of Aroma-Active Compounds and Antioxidant Activity of Cold-Pressed Safflower (*Carthamus tinctorius*) Seed Oils from cvs. Balci and Dincer

**DOI:** 10.1007/s11130-026-01488-y

**Published:** 2026-03-27

**Authors:** Ozlem Kilic-Buyukkurt

**Affiliations:** Department of Food Technology, Kadirli Applied Sciences School, Osmaniye Korkut Ata University, 80760 Osmaniye, Türkiye

**Keywords:** Safflower, *Carthamus tinctorius*, bioactive compounds, volatile compounds, key odorants, olfactometry

## Abstract

Safflower (*Carthamus tinctorius)* is an annual plant in the *Compositae* family and its seeds provide highly valuable edible oil that has been used since ancient times. In the present study, the aroma profile, aroma-active compounds (AACs), antioxidant activities (AA), and total phenolic contents (TPCs) of the cold-pressed safflower oils obtained from the seeds of two common Turkish varieties, Balci and Dincer, were investigated and compared. The samples from the Balci variety demonstrated greater TPC and AA values compared to the samples from Dincer variety. The oil samples from both varieties exhibited similar aroma profiles; however, significant quantitative differences were observed. The samples from the Balci variety (67,509.0 µg/kg) had higher concentrations of aroma compounds compared to the Dincer variety (32,221.9 µg/kg). A total of 28 volatiles, including terpenes, alcohols, aldehydes, aromatic hydrocarbons, esters, and one compound from each of the carboxylic acid, furan, and ketone, groups were detected in the samples from both varieties. Terpenes were the most abundant aroma group in the samples from both varieties, followed by alcohols. In the oil samples from both varieties, the main AACs were 2,3-butanediol (creamy), (*E*)-*β*-caryophyllene (pungent, salted cheese), and an unidentified compound (green, leafy), all exhibiting high flavor dilution (FD) values. The AACs of the cold-pressed safflower seed oils were identified for the first time in the current study.

**Supplementary Information:**

The online version contains supplementary material available at 10.1007/s11130-026-01488-y.

## Introduction

Safflower (*Carthamus tinctorius* L.), also known as false saffron, is an annual herbaceous plant belonging to the *Asteraceae (Compositae)* family [1] and is native to Southern Asia [2]. It is currently cultivated in many countries, with Kazakhstan, Russia, India, Mexico, the United States of America, and Türkiye being the leading producers according to FAOSTAT 2023. In Türkiye, safflower seed production reached approximately 39,000 tons in 2023 [3]. While this represents a relatively small share compared to other oilseed crops, safflower may be considered a strategic alternative for the Turkish economy, particularly in reducing the national vegetable oil deficit through domestic cultivation. The most widely registered safflower cultivars developed by agricultural research institutes in Türkiye include Balci, Dincer, Yekta, Yenice, and Remzibey05 [4].

Safflower can be grown in arid and semi-arid regions due to its high drought and salinity tolerance [5] and is widely used in food, pharmaceutical, textile, and cosmetic industries. Its seeds and colorful petals have long been utilized as food colorants, textile dyes, and in traditional medicine [6]. Safflower seeds are also an important source of edible oil [7]. In recent years, consumer interest in cold-pressed oils has increased due to their perceived health benefits [8]. Cold pressing preserves nutritional components and produces oils with high polyunsaturated fatty acid content and a nutty flavor [2, 7]. Safflower seeds contain numerous bioactive constituents, including phenolic compounds, which exhibit antioxidant, anticancer, anti-inflammatory, anti-atherogenic, and antimelanogenic activities [4]. The safflower seeds possess 15–22% protein and 11–22% fiber [7]. The seeds are also a valuable source of minerals (Cu, Zn, and Mn), vitamins (A, B, and E), and various tocopherols (*α*,* γ*, and *β*) [2]. Therefore, they play a significant role in reducing the risk of various diseases such as cancer, diabetes, cardiovascular diseases, atherosclerosis, and osteoporosis [4].

Food aroma is a key determinant of quality and consumer preference [9] and arises from the interaction of volatile and non-volatile compounds [1]. Volatile compounds, including terpenes, alcohols, aromatic hydrocarbons, aldehydes, and esters, are commonly identified by gas chromatography-mass spectrometry (GC-MS) due to their volatile properties [10]. Certain compounds, known as aroma-active compounds (AACs) or key odorants, play a decisive role in defining food aroma regardless of their concentration [11]. Although present at trace levels (µg/kg-mg/kg), these compounds are readily perceived by the human nose, whereas some abundant volatiles may not contribute to odor perception. The identification of AACs is achieved by GC-MS-olfactometry (GC-MS-O), which combines instrumental analysis with human sensory detection via a sniffing port, allowing for the discrimination of potent odorants from odorless volatiles, which is not possible using conventional detectors that operate regardless of odor thresholds [9]. Safflower seed oils are characterized by oily, citrus, green, herbaceous, pungent, and peppery aroma notes [7], which can vary with cultivar, environmental conditions, cultivation, and processing parameters [1].

The available literature indicates that only a limited number of studies have investigated the aroma profile of cold-pressed safflower oils [7, 8, 12]. To date, only two studies have focused on the odor-active compounds of safflower seed oil. Wang et al. [1] identified odorants based on relative odor value (ROV), while Han et al. [13] used an electronic nose combined with gas chromatography-ion mobility spectrometry (GC-IMS) to determine the key aroma compounds to discriminate adulterated safflower oil samples. However, no study was found on the AACs in safflower seed oils using GC-MS-O. Therefore, this study represents the first comprehensive evaluation of AACs in cold-pressed safflower seed oil employing GC-MS-O. The objectives of the present study were: (i) to identify and quantify the aroma profile and AACs of cold-pressed safflower seed oils from two common Turkish varieties, Balci and Dincer, (ii) to determine the color, total phenolic contents (TPCs) and antioxidant activities (AAs) of these oils, and (iii) to investigate the effect of the type of plant varieties on the aroma compounds, AACs, TPCs, and AA values. The findings of this study may provide valuable insights for future research on the standardization and authentication of safflower oil in food industry applications.

## Materials and Methods

Materials and Methods are available in the online Supplementary Material (Online Resource 1).

## Results and Discussion

### Oil Yields and Color Properties

The oil yields of cold-pressed safflower seeds were 27.60 and 28.18% (dw) for Dincer and Balci, respectively, with no significant difference between cultivars (*p* > 0.05). These values agree with previous reports ranging from 27.76 to 29.2% [6, 7]. The color properties of cold-pressed safflower oils from the Balci and Dincer varieties are presented in Online Resource 2. Safflower oil is generally described as having a pale to golden yellow color, particularly when obtained from unroasted seeds [7, 14, 15]. In this study, the L*, a*, and b* values of oils from the Balci variety were 30.65, 1.28, and 36.08, respectively, whereas those from the Dincer variety were 32.65, -0.26, and 12.61. Chroma (*C*) and hue angle (*h°*) were calculated to further characterize color; Balci showed higher saturation (36.10) than Dincer (12.61), while *h°* values (87.97° and 91.17°, respectively) indicated both oils remained within the yellow-dominant color space. The statistical evaluation of the color parameters (L*, a*, b*, *C* and *h°*) using the t-test analysis showed significant differences (*p* < 0.01) between the two varieties across all color parameters. The higher L* value of Dincer oils indicated a lighter color compared to Balci oils, while the a* and b* values suggested that Balci oils exhibited a more reddish-yellow hue, whereas Dincer oils appeared less red and more greenish. These differences are consistent with previous report showing that seed oil color varies with seed type and extraction conditions [15]. Reported color values for cold-pressed safflower oil from the Dincer (L* 34.05, a* 1.33, b* 30.08) [7] showed partial agreement with the present findings, with similarity observed mainly in L* values.

## Total Phenolic Content and Antioxidant Activities

The total phenolic content (TPC) and antioxidant activity (AA) results of the cold-pressed safflower oil samples from the Balci and Dincer varieties are also presented in Online Resource 2. The TPC values of the Balci and Dincer varieties were 386.3 mg GAE/kg and 246.3 mg GAE/kg, respectively. According to the t-test results, the safflower varieties had a statistically significant effect on the TPCs of the samples (*p* < 0.01). The results indicated that the oil samples from the Balci variety contained higher TPC compared to the Dincer variety. In a previous study by Aydeniz et al. [7], the TPC of the cold-pressed safflower oil of the Dincer variety was determined as 2,616.1 µg GAE/100 g, indicating a lower level than our findings. A previous study mentioned that the TPC values of the safflower oils from different origins (Morocco, Spain, and India) ranged between 79.5 and 143.7 mg/kg [16]. In another study, the TPCs of the oils extracted from different safflower seed varieties cultivated in Egypt was reported to range between 19.9 and 22.8 mg GAE/100 g oil [14]. Consistent with our results, Günç Ergönül and Aksoylu Özbek [4] reported TPCs of 337.6 and 435.0 mg GAE/kg for cold-pressed Balci and Dincer oils. It was reported that TPC levels are mainly influenced by extraction solvent polarity, variety, climate, harvest time, and storage.

The AA values of the cold-pressed safflower oil samples, evaluated using two distinct methods of DPPH and ABTS, are presented in Online Resource 2. According to the DPPH method, the AAs of the cold-pressed safflower oils from the Balci and Dincer varieties were 594.0 and 184.0 µmol Trolox/kg, respectively while based on the ABTS method, they were 1191.4 and 584.3 µmol Trolox/kg, respectively. The statistical analysis revealed that the varieties had a significant effect on the AA values (*p* < 0.01). Comparison of the data indicated that the Balci variety exhibited higher AA compared to the Dincer variety in both DPPH and ABTS, consistent with the differences observed in the TPCs. A previous study reported TPC values of 289.2-412.8 mg GAE/kg and AA values of 38.9–68.9% for safflower oils from different genotypes [17]. The lack of direct correlation between TPC and DPPH may result from the specific antioxidant activity of individual phenols or interference from non-phenolic substances. Günç Ergönül and Aksoylu Özbek [4] noted that such discrepancies can arise from differing phenolic compositions and oilseed pigments. Phenolic profiles of safflower seeds and oils can vary with variety, maturity, climate, cultivation, and processing [16, 17]. As both oils were obtained under identical cold-pressing conditions, the observed variations in polyphenol content and antioxidant activity may be attributed to the safflower varieties.

## Volatile Composition of the Cold-Pressed Safflower Oils

A total of 28 volatiles were profiled in all safflower oil samples in the current study (Online Resource 3). A total of 27 aroma compounds were identified in the oil samples from the Balci variety seeds including ten alcohols, six terpenes, four aromatic hydrocarbons, two aldehydes, two esters, one ketone, one furan and one carboxylic acid while the oil samples from the Dincer variety seeds contained 23 aroma compounds including nine alcohols, five terpenes, three aromatic hydrocarbons, two esters, two aldehydes, one furan, and one carboxylic acid. The total amount of volatiles was 67,509.0 µg/kg in the oils from the Balci variety while the oils from the Dincer variety had a total quantity of 32,221.9 µg/kg (Online Resource 3). As can be seen, although the oils from both varieties exhibited similar aroma profiles, the total concentration of the volatiles was significantly higher in the oils from the Balci variety compared to the Dincer variety. The statistical analysis revealed that the variety had a significant (*p* < 0.01) effect on the total aroma concentrations of the cold-pressed safflower oil. In an earlier study comparing the aroma profiles of safflower oils from different plant varieties, the differences among the aroma compounds were related not only to variety, but also to climate, geographical location and different environmental conditions [1].

## Terpenes

Terpenes were found to be the dominant aroma groups in the cold-pressed oils from both of the safflower varieties (Online Resource 3). Yin et al. [18] stated that terpenes were the main aroma group in cold-pressed sunflower oil, consistent with our finding. In the present study, six and five terpenes were detected in the oils samples from the Balci and Dincer varieties, with total concentrations of 39,375.2 and 15,838.6 µg/kg, respectively, indicating that the Balci variety had a significantly higher overall terpene content compared to the Dincer variety. The oil samples from both varieties contained *α*-pinene, dl-limonene, *γ*-terpinene, and *p*-cymene. The oil samples from the Balci variety additionally had *β*-myrcene and (*E*)-*β*-caryophyllene while the Dincer variety uniquely contained *α*-phellandrene. A previous study similarly reported the presence of *α*-phellandrene in cold-pressed safflower seed oil from the Dincer variety [7]. Among the terpenes in the current study, dl-limonene exhibited the highest concentration in both cold-pressed safflower oil samples. dl-Limonene comprised 83% and 73% of the total terpene fraction in Balci and Dincer, respectively. It also represented 48% and 36% of the total aroma concentration, confirming its dominance in the volatile profile of both varieties. dl-Limonene is a naturally occurring monoterpenoid in safflower oils as indicated by Wang et al. [1]. In an earlier study examining the aroma profile of the cold-pressed safflower oils obtained from Dincer variety, similar to our findings, *α*-pinene, dl-limonene, *p*-cymene, and (*E*)-*β*-caryophyllene were detected [7]. After dl-limonene, *γ*-terpinene was the most abundant compound in the Balci variety (34,60.02 µg/kg) whereas *p-*cymene was the most common in the Dincer variety (1,673.17 µg/kg). *β*-Myrcene and *(E)-β-*caryophyllene were detected at low levels in the Balci variety, whereas *α-*phellandrene was detected in low amounts in the Dincer variety.

### Alcohols

Alcohols, following terpenes, were the dominant aroma group (Online Resource 3). Higher numbers of alcohols were detected compared to the terpenes but their concentrations were lower. In a previous study on the cold-pressed safflower oils from China, alcohols were also abundant and contributed significantly to aroma [1]. Ten and nine alcohols were identified in Balci and Dincer oils, with total concentrations of 14,535.2 and 9,304.0 µg/kg, respectively, with Balci oils containing higher amounts. 2-Methyl-2-butanol, 3-penten-2-ol, 3-hexanol, 2-hexanol, diacetone alcohol, 1-hexanol, 2,3-butanediol, benzyl alcohol, and phenethyl alcohol were present in the oil samples from both varieties, while 1-heptanol was detected only in the oils from the Balci variety. 2-Methyl-2-butanol was the dominant alcohol (5054.78 µg/kg) in the oils from the Balci variety, followed by 1-hexanol (4140.50 µg/kg). The same compound was also the most abundant alcohol in the Dincer variety (4677.31 µg/kg) with 2-hexanol (1017.40 µg/kg) as the second most abundant constituent. Alcohols present in edible oils are mainly formed through the reduction of carbonyl compounds or the degradation of fatty acid hydroperoxides, both of which occur during lipid oxidation [10]. The lipid oxygenase and alcohol dehydrogenase enzyme activity may be the cause of comparatively elevated n-hexanol levels [1]. In a previous GC-IMS study on safflower oil adulteration [13], 2,3-butanediol was found in non-adulterated cold-pressed oil, consistent with our results. 2-Methyl-2-butanol, 3-penten-2-ol, 2-hexanol, 2,3-butanediol, and benzyl alcohol were also detected in argan oils [19], while phenylethyl alcohol was reported in both safflower [7] and argan oils [19].

## Aromatic Hydrocarbons

Four aromatic hydrocarbons were identified in the oil samples from Balci variety, methylbenzene, *p*-xylene, *o*-xylene, and styrene (Online Resource 3). The oil samples from the Dincer variety contained the same compounds except *p*-xylene. Methylbenzene was the predominant aromatic hydrocarbon in both varieties. Wang et al. [1] identified methylbenzene, *p*-xylene, and *o*-xylene in the cold-pressed safflower oils from three different plant varieties from China. Similarly, *p*-xylene was absent in one variety in the same study, which is similar to our results. The total concentrations of the aromatic hydrocarbons were quantified as 6,161.9 and 4,384.8 µg/kg in the oil samples from the Balci and Dincer varieties in the present study. Consistent with the results for the terpenes and alcohols, the Balci variety contained higher total aromatic hydrocarbon levels compared to the Dincer variety.

## Aldehydes

A total of two aldehydes, hexanal and benzaldehyde, were identified in the cold-pressed safflower oil samples from both Balci and Dincer varieties (Online Resource 3). Aldehydes are reported to be formed as degradation products through the secondary cleavage of hydroperoxides [10]. The total aldehyde concentrations were higher in the samples from the Balci variety (2,750.7 µg/kg) compared to the Dincer variety (1,106.9 µg/kg). Hexanal was found to be the most dominant aldehyde in the oil samples from both varieties. In earlier studies, this compound was detected in cold-pressed safflower oils with the highest concentrations [7, 8]. It was indicated to be the most abundant aldehyde in another study [1] on the cold-pressed safflower oils from China. In a subsequent study [13], hexanal and benzaldehyde were also detected in cold-pressed safflower oils from China, in line with results.

### Esters

Esters are the compounds that generally impart fruity odors [11]. Two esters (butyl acetate and ethyl decanoate) were detected in the oil samples from both varieties in the present study (Online Resource 3). Their concentrations were 617.55 and 1,012.09 µg/kg in Balci oils and 509.18 and 774.19 µg/kg in Dincer oils. Ethyl decanoate was higher than butyl acetate in both varieties.

### Other Volatiles

In addition to the compounds above, Balci and Dincer cold-pressed oils contained one carboxylic acid (hexanoic acid) and one furan (2-pentylfuran) (Online Resource 3). Consistent with our findings, 2-pentylfuran has been reported in cold-pressed safflower oils [1], safflower flowers [20], and sunflower oil, where it forms as an oxidation product of linoleic acid and increases with seed roasting [18]. 2-Heptanone, a ketone, was detected only in Balci samples. Ketones form via oxidative breakdown of unsaturated fatty acids [10]. In a study on safflower flower volatiles, 2-heptanone was detected in all samples but at higher concentrations in one variety [20].

### Aroma-Active Compounds

The aroma extract dilution analysis (AEDA) combined with GC-MS-O was applied for the first time to identify the aroma-active compounds (AACs) responsible for the characteristic odor of cold-pressed safflower seed oils from the Balci and Dincer varieties. Online Resource 4 shows the AACs detected in the oil samples along with their flavor dilution (FD) values, odor descriptions, and odor activity values (OAVs). A total of 13 AACs were identified, with FD factors ranging from 2 to 128 and OAVs from 1 to 33. The aromatic extracts of the oil samples from the Balci and Dincer varieties contained 13 and 10 AACs, respectively. The elevated levels of aroma-active compounds in the Balci variety, alongside its high antioxidant capacity, suggest that the aroma profile is primarily shaped by the lipoxygenase pathway and protected from degradation by its potent phenolic profile. The Balci variety was characterized by five alcohols, four terpenes, one ester, one carboxylic acid, and two unidentified compounds, whereas the Dincer variety contained four terpenes, two alcohols, one ester, one carboxylic acid, and two unidentified constituents. The unidentified AACs (LRI: 1,440 and 1,995) were detected by the GC-O system but could not be characterized by the GC-MS, likely due to their very small peak areas and/or the absence of corresponding mass/charge (m/z) ratios in the MS library. However, their high FD factors and consistent odor perception suggest they play a significant role in the aroma profile. This finding demonstrates the advantage of GC-MS-O in detecting potent trace-level odorants that are below the instrumental detection limits of MS. In a study conducted by Wang et al. [1], key odor compounds in safflower oils from different regions were identified using the relative odor activity value. According to their findings, a total of 16 compounds, including 12 aldehydes, two alcohols, one alkene, and one furan, were reported as key odorants (ROV > 1).

In the present study, alcohols were identified as one of the major AAC groups influencing the overall aroma of cold-pressed safflower seed oil. These compounds contribute positively to sensory attributes such as green, bitter, and fruity notes [21]. Five aroma-active alcohols were identified. 2,3-Butanediol and phenethyl alcohol were present in both oils, while 2-methyl-2-butanol, 3-penten-2-ol, and 1-heptanol were found only in Balci. They imparted fresh-camphor, grassy, and herbaceous-green notes (FD 4, 2, and 16), but their contribution to Balci’s overall aroma was minor, especially for 3-penten-2-ol (OAV:2) and 1-heptanol (OAV:1). 3-Penten-2-ol (OAV:2.1) was previously reported as a major aroma contributor in early-harvest Ayvalik olive oil, imparting perfumery and woody notes [22]. In Balci, an unidentified AAC had the highest FD value (128; LRI 1440) with green, leafy odors, while in Dincer, 2,3-butanediol had the highest FD (64) and contributed creamy, sweetish notes (OAV:6). This compound has been reported to impart fruity aromas to raw and freeze-dried jujube [23] and fruity-creamy notes to soy sauce [24]. In the present study, phenethyl alcohol was identified as a key odorant, contributing floral and rose-like aromas to oils from both varieties. Consistent with our study, Pu et al. [24] reported phenethyl alcohol as an aroma-active compound in soy sauce, contributing floral, and rose-like odors. Formed via phenylalanine catabolism, it contributes floral aromas to various foods [25]. In our study, phenethyl alcohol had FD values of 16 and 4 in Balci and Dincer, but its overall aroma contribution was limited due to low OAVs (Balci: 2; Dincer: <1).

Terpenes were the second most influential AAC group affecting the aroma of cold-pressed safflower seed oils (Online Resource 4). Four terpenes were identified: dl-Limonene, *γ*-terpinene, *p*-cymene, and (*E*)-*β*- caryophyllene. Among these, (*E*)-*β*-caryophyllene was the most dominant terpene in both varieties, exhibiting pungent and salted cheese-like odors with FD values of 64 and 32 for the Balci and Dincer varieties, respectively. Although it was not detected by GC-MS in Dincer samples due to its low concentration, its presence was inferred from the detection of the same odor at the corresponding retention time during GC-O analysis. This demonstrates that the compound exists in the Dincer variety at a level that is chemically below the MS detection limit but sensorially significant.

This compound showed the highest FD value among terpenes and relatively high OAVs in the Balci variety. Following *(E)-β*-caryophyllene, *γ*-terpinene showed the highest FD values among terpenes in both varieties, contributing fresh and herbaceous odors with FD values of 32 and 16 and OAVs of 10 and 3 in the Balci and Dincer varieties, respectively. This compound has also been reported as an AAC in cold-pressed sunflower oil, imparting woody and fatty notes [18]. In addition, dl-limonene (citrus, orange-like) and *p*-cymene (woody, fatty) contributed to the typical aroma of safflower oils, with FD values of 8 and 16 in the Balci and Dincer varieties. Notably, dl-limonene in the Balci variety (OAV: 33) and *p*-cymene in the Dincer variety (OAV: 17) exhibited the highest OAVs, indicating their major contribution to the overall aroma. Previous studies have reported limonene as a major odor-active compound in safflower oils from different regions [1] and in roasted sunflower oil [18], imparting citrus and lemon-like odors, consistent with the present findings. Högnadóttir and Rouseff [26] demonstrated that *γ*-terpinene and *β-*caryophyllene contribute terpenic and musty odors, respectively, in orange oil. Similarly, limonene, *γ-*terpinene, and *p*-cymene have been identified as aroma-active compounds in raw and frozen jujube [23].

The other key odorants included an ester (ethyl decanoate) and a carboxylic acid (hexanoic acid) in the oil samples from both varieties (Online Resource 4). Esters are known to contribute desirable fruity and floral odors to foods [25]. In the current study, ethyl decanoate had the FD values of 16 and 4 for the Balci and Dincer varieties, respectively, and imparted fruity and sweet odors. Consistent with the present findings, Jia et al. [27] reported that this compound contributed fruity and grape-like odors to camellia oil. Hexanoic acid, on the other hand, contributed buttery and salty odors to the overall aroma, with FD values of 32 and 8 in the Balci and Dincer varieties, respectively. Ethyl decanoate exhibited OAVs of 5 and 4 in the Balci and Dincer varieties, indicating a perceptible but less dominant contribution to the overall aroma compared to the main odorants that exhibited significantly higher FD factors and OAVs. In contrast, hexanoic acid had an OAV of 2 in Balci and < 1 in Dincer, indicating low contribution in the former and negligible in the latter. Consistent with this, it was identified as an AAC in Moroccan argan oil (FD 8.0, OAV 1.8) with a cheesy odor [19] and also reported to contribute oily notes in olive oil [28].

Key AACs were identified using combined FD factors and OAVs, focusing on compounds with FD ≥ 8 and OAV > 1. As shown in Online Resource 4, dl-limonene, *γ*-terpinene, *p*-cymene, and 2,3-butanediol met both criteria across samples, indicating their roles as key AACs in both varieties. Compounds with high FD but OAV < 1 (*e.g*., hexanoic acid or phenethyl alcohol in Dincer) were considered for sensory contribution but not as primary AACs. This approach integrates sensory sensitivity (FD) and quantitative relevance (OAV). Aydeniz et al. [7] reported that cold-pressed safflower oils produced using different processing methods contained compounds associated with oily, buttery, citrus, fruity, green, herbaceous, woody, pungent, and bitter sensory attributes. Previous studies have indicated that the growing region and plant variety are major factors influencing aroma and AAC profiles [1, 7]. To the best of the author’s knowledge, this is the first study to investigate the key odorants of cold-pressed safflower seed oils from different varieties using GC-MS-O.

## Conclusions

This study is the first investigation to examine the aroma and AAC profiles of cold-pressed safflower oils from two different varieties (Balci and Dincer) using GC-MS-O. The results showed that the safflower variety significantly influenced the color, AA, and TPC of the oil samples. The Balci variety exhibited higher TPCs and AAs than the Dincer variety. The volatile profiles of oil samples from Balci and Dincer varieties were characterized in detail using the GC-MS with purge and trap extraction method. A total of 28 volatile substances were identified in both varieties, including terpenes, alcohols, aldehydes, aromatic hydrocarbons, esters, and one compound from each of the carboxylic acid, furan, and ketone groups. Regarding the AACs, a total of 13 constituents, including alcohols, terpenes, esters, carboxylic acids, and two unidentified substances, were determined in the samples using GC-MS-O. The differences in the volatile profile and concentrations of the oil samples can be attributed to the difference in varieties. The highest concentrations detected for both varieties belonged to terpenes, followed by alcohols. Meanwhile, 2,3-butanediol, (*E*)-*β*-caryophyllene, and an unidentified compound were detected as major AACs contributing creamy, pungent, and green-leafy aromas. The findings of the present study revealed that GC-MS-O can offer valuable scientific support for the detection and evaluation of the authenticity of the AACs in cold-pressed safflower oils. Further research is needed to clarify the determinants of AAC composition in safflower oil. However, it should be noted that this study is limited to the analysis of two specific cultivars. Future research covering a broader range of varieties, diverse environmental conditions, and harvest effects is needed to clarify the determinants of AACs and enhance the generalizability of these findings. Furthermore, these results should be related to quantitative descriptive analysis and assess the stability of key odorants during storage. Industrially, these compounds may serve as quality and authentication markers, supporting consumer acceptance and shelf-life optimization in food applications.

## Supplementary Information

Below is the link to the electronic supplementary material.

Supplementary Material 1

Supplementary Material 2

Supplementary Material 3

Supplementary Material 4

## Data Availability

The data generated and/or analyzed during the current study are available from the corresponding author on reasonable request.

## Abbreviations

AACs: Aroma-active compounds
AAs: Antioxidant activities
ABTS: 2,2’-azinobis-(3-ethylbenzothiazoline-6-sulfonic acid)
DPPH: 1,1-diphenyl-2-picrylhydrazyl
FD: Flavor dilution
GC-MS-O: Gas chromatography-mass spectrometry-olfactometry
OAV: Odor activity value
ROV: Relative odor value
TPCs: Total phenolic contents
