# Supplementary material for: Characterization of Aroma-Active Compounds and Antioxidant Activity of Cold-Pressed Safflower (Carthamus tinctorius) Seed Oils from cvs. Balci and Dincer
Source: Plant Foods Hum Nutr. 2026 Mar 27;81(2):40. doi: 10.1007/s11130-026-01488-y (PMC13021708; doi:10.1007/s11130-026-01488-y)
Supplement: Supplementary file 1 — Supplementary Material 1 [file 11130_2026_1488_MOESM1_ESM.pdf]

ESM 1 (Online Resource 1): Supplemental Materials and Methods

Characterization of Aroma-Active Compounds and Antioxidant Activity of Cold-Pressed Safflower (*Carthamus tinctorius*) Seed Oils from cvs. Balci and Dincer

Ozlem Kilic-Buyukkurt[1]

[1] **Department of Food Technology, Kadirli Applied Sciences School, Osmaniye Korkut Ata University, 80760 Osmaniye, Türkiye**

Correspondence: ozlemkilic@osmaniye.edu.tr, ORCID: 0000-0001-5786-6655

Materials and Methods

Materials

The safflower seeds used in the current study belonging to two locally developed varieties, namely Balci and Dincer, were obtained from the Transitional Zone Agricultural Research Institute, located in the Eskisehir province of Türkiye. These varieties were selected due to their high commercial importance and widespread cultivation as registered domestic varieties in Türkiye. Foreign materials were separated from the seeds and then the seeds were stored in a cool and dry place until the cold-pressing process to obtain oil.

Chemicals

The chemicals utilized in the study, including DPPH (1,1-diphenyl-2-picrylhydrazyl), ABTS (2,2'-azinobis-(3-ethylbenzothiazoline-6-sulfonic acid), Folin–Ciocalteu reagent, gallic acid, potassium persulfate, sodium carbonate, dichloromethane, along with the aroma internal standard (4-nonanol), were of analytical and chromatographic grade and obtained from the Sigma-Aldrich company (St. Louis, USA). The distilled water used in the study was purified through a Millipore-Q system (Millipore Corp., Saint-Quentin, France).

Cold pressing process

The cleaned safflower seeds, which had an initial moisture content of 5%, were pressed directly with a cold pressing machine (Karaerler NF80, Ankara, Türkiye) without further drying. The machine had a 15 mm nozzle and a screw rotational speed of 35 Hz. The temperature of the safflower seed oils was approximately 45°C. Following extraction, the oil was allowed to settle for 24 h and the clear phase was separated by decantation to remove suspended solids. The cold-pressed safflower oil samples were placed into amber colored glass bottles and kept in a cool, dark and dry place until the analyses. The oil yield was expressed as %, w/w of recovered oil to seeds dry weight.

Color properties

The color parameters (*L**, *a**, and *b**) of safflower seed oils were measured using a colorimeter (HunterLab ColorQuest XE, USA). In the CIE Lab color system, *L** indicates lightness (0= black, 100= white), *a** represents the red-green axis, and *b** represents the yellow-blue axis. Additionally, chroma (*C*) and hue angle (*h*°) were calculated to provide a more detailed description of the color attributes [1].

Analysis of phenolic compounds

Extraction of phenolic compounds

Phenolic compounds were extracted according to the International Olive Oil Council (IOOC) official procedure (COI/T.20/Doc No:29) with minor modifications [2]. Briefly, 2 g of safflower oil was mixed with 5 mL of methanol-water (80:20, v/v), vortexed for 1 min, and centrifuged at 5000 rpm for 15 min. The supernatant was collected and stored at 4°C until analysis.

Analysis of the antioxidant activity and total phenolic content

The antioxidant activities (AAs) of the safflower seed oil samples were evaluated using two different assays including, DPPH and ABTS. For the DPPH analysis, a 0.1 mL aliquot of the extract was combined with 3.9 mL of a 0.1 mM methanolic DPPH solution. Following a 30-minute incubation period at 25°C in the dark, the absorbance was recorded at 515 nm using a spectrophotometer (PG Instruments, T60 Visible, UK). In the ABTS assay, the radical cation solution was prepared by reacting 7 mM ABTS with 2.5 mM potassium persulfate in the dark for 16 hours. This mixture was then diluted with 80% methanol to reach an absorbance of 0.70 ± 0.01 at 734 nm. For the measurement, 0.1 mL of the extract was added to 3.9 mL of this solution. The mixture was incubated for 30 minutes in the dark, and the absorbance was measured at 734 nm. The AA values were calculated using a Trolox standard calibration curve and expressed as µmol Trolox/kg. The total phenolic contents (TPCs) of the oil samples were measured via the Folin-Ciocalteu analysis, with the data quantified from a gallic acid calibration curve (50-500 mg/kg) and expressed as mg GAE/kg [3].

Analysis of the volatile compounds

Extraction of the volatile compounds

Volatile compounds in safflower seed oils were isolated using the purge-and-trap extraction method according to Kilic Buyukkurt et al. [4]. This method employed a nitrogen source controlled by a flow-meter (LZT 4-M, Union-Tek Instrument, China). The needle of the nitrogen source and the cartridge were installed through the septum to purge

and trap the aroma substances. Three grams from the cold-pressed oil samples were placed into a 20 mL vial and then 2.5 µL internal standard (4-nonanol) was added. The sample was pre-incubated at 60°C for 10 min before nitrogen gas was applied. The extraction process was performed under 500 mL/min nitrogen gas flow for 2 hours at 60°C. After the extraction, the aroma compounds retained by the cartridge were taken by using 6 mL of dichloromethane. Then, the obtained aroma extract was evaporated with a Vigreux distillation column to 0.5 mL at 50°C. All extracts were stored in insert vials at -20°C until the injection to gas chromatography (GC).

GC-FID and GC-MS analysis of the volatile compounds

GC analyses were performed using a Shimadzu Nexis GC-2030 equipped with a flame ionization detector (FID) and coupled to a Shimadzu GC-MS-QP2020 NX system with an integrated olfactometric port (Kyoto, Japan). A Dean's switch was used to split the column effluent equally (1:1:1) among the olfactometric port, FID, and MS detectors. Volatile compounds were separated on a DB-Wax column (30 m × 0.25 mm i.d. × 0.5 µm; J&W Scientific, USA). The oven temperature was programmed from 50 to 250 °C at 4 °C/min, with a final hold at 250 °C for 10 min. A 3 µL sample was injected in pulsed splitless mode (40 psi, 0.5 min). The injector and FID temperatures were set at 270 and 280 °C, respectively, and helium was used as the carrier gas at a flow rate of 1.5 mL/min. The MS was operated under electron impact ionization (70 eV), scanning from m/z 30–300 at 2.0 scans/s, with interface, ion source, and quadrupole temperatures of 250, 180, and 150°C, respectively. Volatile compounds were identified by comparing their retention indices (RI) and mass spectra on the DB-Wax column with those in commercial libraries (Wiley 10, NIST-11, and Flavor.2L) and the instrument's internal library created from previous laboratory studies. Identification of certain compounds was verified through the injection of chemical standards and their retention indices were determined using n-alkane (C_8-C_{32}) series. Quantification was performed using 4-nonanol as the internal standard. Response factors were determined relative to the internal standard, and peak areas were adjusted accordingly. Results were expressed as mean ± standard deviation of replicated GC analyses. The final concentrations were calculated using the following formula:

$$C_i = (A_i/A_{std}) \times C_{std} \times RF \times CF$$

where, C_i: Concentration of the aroma compound; C_{std}: Concentration of the internal standard (41.5 mg/kg); A_i: The peak area of the aroma compound; A_{std}: The peak area of the internal standard; RF: Response factor; and CF: Calculation factor [4].

Determination of the aroma-active compounds

The aroma extract dilution analysis (AEDA) procedure was performed to identify the aroma-active compounds (AACs) of the oil samples. The concentrated aromatic extracts were serially diluted with dichloromethane by various dilution ratios (1:1, 1:2, 1:4, ..., 1:128) and then subsequently analyzed by the GC-MS-O. The analyses were conducted by a panel of two trained assessors, each with prior experience in aroma evaluation. The dilution process was continued until the odor was no longer perceptible and at that point, the analysis was concluded. The flavor dilution (FD) factor of an odorant corresponds to the highest dilution at which it remains detectable at the sniffing port [3].

Determination of the odor activity values

The odor activity values (OAVs) were calculated to assess the effect of aroma compounds on the overall aroma of the cold-pressed safflower oil samples. The OAV values were determined by dividing the concentration of each aroma substance by its odor threshold (OT) value reported in the literature for oil matrices [5-10]. Compounds with the OAVs greater than 1.0 were considered as being capable of contributing individually to the overall aroma of the oil sample [7].

Statistical data analysis

The data of the present study were subjected to an independent t-test at a 99% confidence interval using SPSS 20.0 (SPSS Inc., Chicago, IL, USA) to determine the statistical significance of the differences between the means. The numerical data were expressed as the mean values and standard deviations.

References

1. Guclu G, Keser D, Kelebek H et al (2021) Impact of production and drying methods on the volatile and phenolic characteristics of fresh and powdered sweet red peppers. Food Chem. https://doi.org/10.1016/j.foodchem.2020.128129
2. International Olive Oil Council (IOOC) (2017) COI/T.20/Doc No 29-Rev-1– Determination of Biophenols in Olive Oils by HPLC. https://www.internationaloliveoil.org/wp-content/uploads/2019/11/COI-T.20-Doc.-No-29-Rev-1-2017.pdf. Accessed 1 Aug 2025
3. Salman E, Guclu G, Pehlivan ZY, Kelebek H, Selli S (2025) Changes in volatile, key odorants and bioactive properties of pomegranate juice during processing into concentrate. Food Chem. https://doi.org/10.1016/j.foodchem.2025.142856
4. Kilic Buyukkurt O, Guclu G, Kelebek H, Selli S (2025) Characterization of the key odorants of mastic gum (*Pistacia lentiscus* var. Chia) from two different countries. Appl Sci. https://doi.org/10.3390/app15105329

5. Sevindik O, Kelebek H, Rombolà AD, Selli S (2022) Grape seed oil volatiles and odour activity values: a comparison with Turkish and Italian cultivars and extraction methods. J Food Sci Technol. https://doi.org/10.1007/s13197-021-05212-3
6. Jia X, Zhou Q, Huang D et al (2024) Insight into the comparison of key aroma-active compounds between camellia oils from different processing technology. Food Chem. https://doi.org/10.1016/j.foodchem.2023.137090
7. Kalua C, Allen M, Bedgood D, Bishop A, Prenzler P, Robards K (2007) Olive oil volatile compounds, flavour development and quality: A critical review. Food Chem. https://doi.org/10.1016/j.foodchem.2005.09.059
8. Narbona E, García-García E, Vázquez-Araújo L, Carbonell-Barrachina AA (2010) Volatile composition of functional 'a la Piedra' turrón with propolis. Int J Food Sci Technol. https://doi.org/10.1111/j.1365-2621.2009.02167.x
9. Multari S, Vall AM, Yang B, Suomela JP (2018) Effects of aromatic herb flavoring on carotenoids and volatile compounds in edible oil from blue sweet lupin (*Lupinus angustifolius*). Eur J Lipid Sci Technol. https://doi.org/10.1002/ejlt.201800227
10. Giri A, Osako K, Ohshima T (2010) Identification and characterization of headspace volatiles of fish miso, a Japanese fish meat based fermented paste, with special emphasis on effect of fish species and meat washing. Food Chem. https://doi.org/10.1016/j.foodchem.2009.10.036
