# Supplementary material for: Characterization of Aroma-Active Compounds and Antioxidant Activity of Cold-Pressed Safflower (Carthamus tinctorius) Seed Oils from cvs. Balci and Dincer
Source: Plant Foods Hum Nutr. 2026 Mar 27;81(2):40. doi: 10.1007/s11130-026-01488-y (PMC13021708; doi:10.1007/s11130-026-01488-y)
Supplement: Supplementary file 2 — Supplementary Material 2 [file 11130_2026_1488_MOESM2_ESM.pdf]

ESM 2 (Online Resource 2): Supplemental Table S1

Characterization of Aroma-Active Compounds and Antioxidant Activity of Cold-Pressed Safflower (*Carthamus tinctorius*) Seed Oils from cvs. Balci and Dincer

Ozlem Kilic-Buyukkurt[1]

[1] Department of Food Technology, Kadirli Applied Sciences School, Osmaniye Korkut Ata University, 80760 Osmaniye, Türkiye

Correspondence: ozlemkilic@osmaniye.edu.tr, ORCID: 0000-0001-5786-6655

ESM 2 (Online Resource 2): Table S1 Color properties, total phenolic contents (TPCs), and antioxidant activities (AAs) of the cold-pressed safflower seed oil samples from the Balci and Dincer varieties

	**Balci**	**Dincer**	***p*[1]**
**Color properties**			
*Color L**	30.65 ± 0.01	32.65 ± 0.04	**
*Color a**	1.28 ± 0.06	-0.26 ± 0.04	**
*Color b**	36.08 ± 0.10	12.61 ± 0.04	**
C	36.10 ± 0.10	12.61 ± 0.04	**
h°	87.97 ± 0.09	91.17 ± 0.16	**
**TPC (mg GAE/kg)**	386.3 ± 5.00	246.3 ± 1.67	**
**AA, DPPH (µmol Trolox/kg)**	594.0 ± 14.14	184.0 ± 14.15	**
**AA, ABTS (µmol Trolox/kg)**	1191.4 ± 95.96	584.3 ± 65.66	**

Data are expressed as means ± standard deviations of the three replicates.
[1] Statistical significance according to the t-test results: **: significant at $p<0.01$.
