# Supplementary material for: Characterization of Aroma-Active Compounds and Antioxidant Activity of Cold-Pressed Safflower (Carthamus tinctorius) Seed Oils from cvs. Balci and Dincer
Source: Plant Foods Hum Nutr. 2026 Mar 27;81(2):40. doi: 10.1007/s11130-026-01488-y (PMC13021708; doi:10.1007/s11130-026-01488-y)
Supplement: Supplementary file 3 — Supplementary Material 3 [file 11130_2026_1488_MOESM3_ESM.pdf]

# ESM 3 (Online Resource 3): Supplemental Table S2

# Characterization of Aroma-Active Compounds and Antioxidant Activity of Cold-Pressed Safflower (*Carthamus tinctorius*) Seed Oils from cvs. Balci and Dincer

**Ozlem Kilic-Buyukkurt**[1]

[1] **Department of Food Technology, Kadirli Applied Sciences School, Osmaniye Korkut Ata University, 80760 Osmaniye, Türkiye**

**Correspondence: ozlemkilic@osmaniye.edu.tr, ORCID: 0000-0001-5786-6655**

**ESM 3 (Online Resource 3): Table S1** Aroma profiles of the cold-pressed safflower oil samples from the Balci and Dincer varieties

| No | LRI[1] | Aroma compounds | Concentration (µg/kg)[2] | | Identification[3] | *p*[4] |
|---|---|---|---|---|---|---|
| | | | Balci | Dincer | | |
| | | **Alcohols** | | | | |
| 1 | 997 | 2-Methyl-2-butanol | 5054.78 ± 74.74 | 4677.31 ± 181.95 | LRI, MS, std | ** |
| 2 | 1172 | 3-Penten-2-ol | 999.31 ± 69.69 | 932.28 ± 34.82 | LRI, MS, std | ns |
| 3 | 1238 | 3-Hexanol | 449.68 ± 12.49 | 222.02 ± 9.05 | LRI, MS, std | ** |
| 4 | 1226 | 2-Hexanol | 1336.62 ± 127.00 | 1017.40 ± 52.53 | LRI, MS, std | ** |
| 5 | 1351 | Diacetone alcohol | 681.17 ± 64.37 | 881.72 ± 28.27 | LRI, MS, std | ** |
| 6 | 1380 | 1-Hexanol | 4140.50 ± 147.14 | 613.46 ± 2.27 | LRI, MS, std | ** |
| 7 | 1457 | 1-Heptanol | 163.61 ± 2.74 | nd | LRI, MS, std | ** |
| 8 | 1545 | 2,3-Butanediol | 849.51 ± 81.71 | 574.84 ± 55.65 | LRI, MS, std | ** |
| 9 | 1821 | Benzyl alcohol | 286.89 ± 20.93 | 157.48 ± 10.83 | LRI, MS, std | ** |
| 10 | 1952 | Phenethyl alcohol | 573.08 ± 43.55 | 227.50 ± 15.80 | LRI, MS, std | ** |
| | | **Total** | **14535.2** | **9304.0** | | |
| | | **Terpenes** | | | | |
| 11 | 1043 | *α*-Pinene | 878.98 ± 47.20 | 540.99 ± 0.51 | LRI, MS, std | ** |
| 12 | 1177 | *α*-Phellandrene | nd | 815.33 ± 58.10 | LRI, MS, std | ** |
| 13 | 1189 | dl-Limonene | 32677.01 ± 1769.36 | 11643.99 ± 249.33 | LRI, MS, std | ** |
| 14 | 1273 | *γ*-Terpinene | 3460.02 ± 119.25 | 1165.07 ± 11.28 | LRI, MS, std | ** |
| 15 | 1283 | *p*-Cymene | 832.96 ± 55.20 | 1673.17 ± 20.23 | LRI, MS, std | ** |
| 16 | 1176 | *β*-Myrcene | 830.60 ± 25.70 | nd | LRI, MS, std | ** |
| 17 | 1628 | (*E*)-*β*-Caryophyllene | 695.59 ± 7.16 | nd | LRI, MS, std | ** |
| | | **Total** | **39375.2** | **15838.6** | | |
| | | **Aromatic hydrocarbons** | | | | |
| 18 | 1038 | Methylbenzene | 3309.09 ± 98.68 | 3291.20 ± 110.18 | LRI, MS, tent | ns |
| 19 | 1149 | *p*-Xylene | 333.16 ± 11.72 | nd | LRI, MS, std | ** |
| 20 | 1169 | *o*-Xylene | 970.59 ± 92.58 | 597.17 ± 5.06 | LRI, MS, std | ** |
| 21 | 1260 | Styrene | 1549.03 ± 114.24 | 496.44 ± 43.94 | LRI, MS, tent | ** |

| | | | | | | |
|---|---|---|---|---|---|---|
| | | **Total** | **6161.9** | **4384.8** | | |
| | | **Aldehydes** | | | | |
| 22 | 1081 | Hexanal | 2478.63 ± 84.77 | 855.82 ± 25.17 | LRI, MS, std | ** |
| 23 | 1470 | Benzaldehyde | 272.10 ± 7.69 | 251.11 ± 13.58 | LRI, MS, std | * |
| | | **Total** | **2750.7** | **1106.9** | | |
| | | **Esters** | | | | |
| 24 | 1078 | Butyl acetate | 617.55 ± 21.12 | 509.18 ± 9.95 | LRI, MS, std | ** |
| 25 | 1643 | Ethyl decanoate | 1012.09 ± 11.99 | 774.19 ± 16.04 | LRI, MS, std | ** |
| | | **Total** | **1629.6** | **1283.4** | | |
| | | **Others** | | | | |
| 26 | 1797 | Hexanoic acid | 1683.15 ± 27.55 | 135.58 ± 7.26 | LRI, MS, std | ** |
| 27 | 1249 | 2-Pentylfuran | 623.22 ± 17.33 | 168.63 ± 13.03 | LRI, MS, tent | ** |
| 28 | 1185 | 2-Heptanone | 750.08 ± 38.71 | nd | LRI, MS, std | ** |
| | | **Total** | **3056.5** | **304.2** | | |
| | | **Grand Total** | **67509.0** | **32221.9** | | |

[1]Linear retention index (LRI) was measured on a DB-WAX capillary column.
[2]Concentrations are expressed as μg/kg ± standard deviation (mean of the two replicates); [2]nd: not detected.
[3]: Identification methods: LRI: linear retention index; tent: tentative identification by the MS; std: confirmed with authentic standard compounds.
[4]: Statistical significance was evaluated by the t-test analysis; ns: not significant, *: significant at $p<0.05$, **: significant at $p<0.01$
