# Supplementary material for: Characterization of Aroma-Active Compounds and Antioxidant Activity of Cold-Pressed Safflower (Carthamus tinctorius) Seed Oils from cvs. Balci and Dincer
Source: Plant Foods Hum Nutr. 2026 Mar 27;81(2):40. doi: 10.1007/s11130-026-01488-y (PMC13021708; doi:10.1007/s11130-026-01488-y)
Supplement: Supplementary file 4 — Supplementary Material 4 [file 11130_2026_1488_MOESM4_ESM.pdf]

ESM 4 (Online Resource 4): Supplemental Table S3

Characterization of Aroma-Active Compounds and Antioxidant Activity of Cold-Pressed Safflower (*Carthamus tinctorius*) Seed Oils from cvs. Balci and Dincer

Ozlem Kilic-Buyukkurt[1]

[1] Department of Food Technology, Kadirli Applied Sciences School, Osmaniye Korkut Ata University, 80760 Osmaniye, Türkiye

Correspondence: ozlemkilic@osmaniye.edu.tr, ORCID: 0000-0001-5786-6655

ESM 4 (Online Resource 4): Table S1 Odor descriptions, flavor dilution (FD) values, odor activity values (OAV), and odor thresholds (OT) of the aroma-active compounds (AACs) in the cold-pressed safflower oil samples from the Balci and Dincer varieties

				FD[3]		Odor threshold (µg/L)[4]	OAV[5]		
**No**	**LRI[1]**	**Compounds**	**Odor descriptions[2]**	**Balci**	**Dincer**		**Balci**	**Dincer**	**Ref.**
1	997	2-Methyl-2-butanol	Fresh, camphor	4	-	-	-	-	
2	1172	3-Penten-2-ol	Green, grassy	2	-	400	2	-	[7]
3	1189	dl-Limonene	Citrus, orange like	8	16	1000	33	12	[8]
4	1273	*γ*-Terpinene	Fresh, herbaceous	32	16	350	10	3	[9]
5	1283	*p*-Cymene	Woody, fatty	8	16	100	8	17	[9]
6	1440	Unidentified	Green, leafy	128	32	-	-	-	
7	1457	1-Heptanol	Herbaceous, green	16	-	200	1	-	[6]
8	1545	2,3-Butanediol	Creamy, sweetish	64	64	95.1	9	6	[10]
9	1628	(*E*)-*β*-Caryophyllene	Pungent, salted cheese	64	32	64	11	-	[8]
10	1643	Ethyl decanoate	Fruity, sweetish	16	4	200	5	4	[5]
11	1797	Hexanoic acid	Buttery, salty	32	8	700	2	<1	[7]
12	1952	Phenethyl alcohol	Floral, rose	16	4	1100	1	<1	[5]
13	1995	Unidentified	Floral, sweetish	16	4	-	-	-	

[1]LRI: Linear retention index which indicates the retention indices determined on the DB-WAX capillary column.
[2]Odor descriptions as perceived by the panelists via olfactometry.
[3]FD: Flavor dilution factor: The highest dilution of the extract where an odorant was perceived.
[4]OT: Odor threshold values in the oil samples (µg/L) which were obtained from the literature [References are cited in the Supplementary Material 1: 5-10].
[5]OAV: Odor activity values calculated as the compound concentration divided by its odor threshold.
